# The mediating role of work engagement: A survey data on organizational citizenship behavior

**DOI:** 10.1016/j.dib.2022.108243

**Published:** 2022-05-06

**Authors:** Ahmad Rizki Sridadi, Anis Eliyana, Desynta Rahmawati Gunawan, Muhammad Danang Kurniawan, Alvin Permana Emur, Zaleha Yazid

**Affiliations:** a Universitas Airlangga, Indonesia; b PT Usaha Mulia Digital Indonesia (PT UMDI), Indonesia; c Universiti Kebangsaan Malaysia, Malaysia

**Keywords:** Psychological capital, Transformational leadership, Work engagement, Organizational citizenship behavior, Secure working environments, Effective institutions

## Abstract

This dataset shows the results of research on factors that influence organizational citizenship behavior (OCB). Other variables included in the dataset are Psychological Capital (PC), Transformational Leadership (TL), and Work Engagement (WE). The dataset includes 156 respondents who are client counsellors (CC) in 19 correctional institutions throughout Indonesia. Data was collected with the help of an online questionnaire which was carried out in 3 waves. The dataset in this article also applies multi-group analysis to validate the drivers of organizational citizenship behavior in doing work which is divided into male and female employees.

## Specifications Table

**Table:** 

Subject	Organizational behavior
Specific subject area	Management, Human Resource Management, Human Capital
Type of data	Tables and Figures
How data were acquired	Survey Questionnaire. Data was collected using a five-point Likert scale. All valid samples were processed with SPSS and Amos.
Data format	Raw, analyzed
Parameters for data collection	The dataset uses the sampling method with purposive sampling technique through the criteria of officers who interact directly with the inmates, and whose duty is to provide guidance and counselling.
Description of data collection	Primary data sources were collected from the distribution of online questionnaires (Google Form) involving 3 waves of data collection. The three waves of data were collected at an interval of 30 days in 2021.
Data source location	Correctional institutions throughout Indonesia, 6° North Latitude −11° South Latitude & 95° East Longitude - 141° East Longitude
Data Accessibility	Repository name: Mendeley data, Data identification number: 10.17632/fdzs82y645.2

## Value of the Data

• The dataset provides evidence how organizational citizenship behavior create a more effective work environment.
• The dataset provides insight that individual behavior and perceptions, which are the main drivers of extra behavior in organizations, can be support success.
• The dataset is useful for predicting the psychological condition, leadership style, attitude, and behavior of employees on discretionary behavior.
• The dataset can be used to explain how employee involvement in work can support realizing the right psychological and leadership style for employees towards discretionary behavior in the workplace.
• The dataset is important for policy implementation and can encourage coordination of activities among team members and in generating a peer-to-peer environment in organizational learning activities.

## 1 Data Description

The data set includes questions related to four constructs through psychological capital adopted from research [1], then for transformational leadership adopted from research [2], then to measure work engagement adopted from research [3], and to measure organizational citizenship behavior adopted from research [4]. Measurements on the indicators used using a Likert scale 1 = strongly disagree, 2 = disagree, 3 = neutral, 4 = agree, and 5 = strongly agree. The scale is used to measure indicators that show respondents' responses to the statements contained in the questionnaire.

Counsellor is part of the correctional officers whose job is to guide prisoners so that they can re-integrate socially into the community while the counselling process itself starts 1 year before the incarceration term ends. The total population of counsellors in Indonesia is 1565 officers, however the present study focuses only on counsellors for terrorism convict. Hence, the sample size is 156 officers. The entire demographic characteristics of the respondents in this study are illustrated in Table 1. The characteristics of the respondents will be described based on gender, marital status, age, tenure, entry path, type of position, last education degree.

**Table 1:** Respondents' profile.

Respondents' profile	Description	Frequency	Percentage
Gender	Male	94	60.3
	Female	62	39.7

Marital Status	Not Married	22	14.1
	Married	127	81.4
	Widowed	7	4.5

Age	20–30 Years old	32	20.5
	31–40 Years old	67	42.9
	41–50 Years old	30	19.2
	>50 Years old	27	17.3

Tenure	1–2 Years old	24	15.4
	3–4 Years old	16	10.3
	5–6 Years old	2	1.3
	7–8 Years old	2	1.3
	> 8 Years old	112	71.8

Entrance Path for	Civil Servant General Path	150	96.2
	Civil Servant Official School	6	3.8

Types	Functional	156	100.0

Last Education Degree	SHS/VHS/Similar	25	16.0
	Diploma	5	3.2
	Bachelor Degree	110	70.5
	Master Degree	16	10.3

*Note:* Six demographic variables are coded in the data as Gender (1-Female, 2-Male), Marital Status (1- Not Married, 2- Married, 3- Widowed/Widow), Age (1–20–30, 2 −31–40, 3–41–50, 4->50), Tenure (1–1–2 years, 2–3–4 years, 3–5–6 years, 4–7–8 years, ->8 years) Entrance Path (1- through general selection, 2- through civil servant service schools), Type of Position (1-Structural), Education Degree (1- Senior High School, 2-Diploma, 3-Bachelor, 4-Master, 5-Doctor).

Table 2 presents the results of data analysis based on respondents' answers to the questionnaire. The average value of respondents' responses to each indicator and the overall value of each research variable are also shown in Table 2.

**Table 2:** Descriptive statistics criterion.

*Psychological Capital*							

	Likert scale percentage						
Indicator	SD	D	N	A	SA	Mean	SD

PC1	1.9	9.0	22.4	41.7	25.0	3.79	0.98
PC2	1.3	8.3	21.2	41.7	27.6	3.86	0.96
PC3	0.0	8.3	14.7	48.1	28.8	3.97	0.88
PC4	0.6	4.5	17.3	45.5	32.1	4.04	0.86
PC5	0.0	7.7	17.9	49.4	25.0	3.92	0.86
PC6	0.0	5.1	21.8	42.9	30.1	3.98	0.85
PC7	0.0	0.6	17.9	57.7	23.7	4.04	0.67
PC8	0.0	1.3	26.3	53.2	19.2	3.90	0.71
PC9	0.6	3.8	34.6	43.6	17.3	3.73	0.81
PC10	0.0	1.9	23.1	50.0	25.0	3.98	0.75
PC11	0.0	2.6	25.6	52.6	19.2	3.88	0.74
PC12	1.3	3.2	34.6	44.9	16.0	3.71	0.82

Variable Mean						3.90	0.82

*Transformational Leadership*							

TL1	1.3	3.8	29.5	43.6	21.8	3.81	0.87
TL2	0.6	3.8	31.4	41.0	23.1	3.82	0.85
TL3	0.6	3.2	26.9	46.8	22.4	3.87	0.82
TL4	0.6	2.6	25.0	45.5	26.3	3.94	0.82
TL5	0.0	1.3	15.4	55.1	28.2	4.10	0.69
TL6	0.0	0.0	14.7	51.9	33.3	4.19	0.67
TL7	0.0	0.0	19.9	47.4	32.7	4.13	0.72
TL8	0.0	0.0	21.2	48.7	30.1	4.09	0.71
TL9	0.0	0.0	15.4	51.3	33.3	4.18	0.68
TL10	0.0	0.0	14.1	53.8	32.1	4.18	0.66
TL11	0.0	0.6	25.6	46.2	27.6	4.01	0.75
TL12	0.6	4.5	32.1	40.4	22.4	3.79	0.86
TL13	9.0	20.5	29.5	23.7	17.3	3.20	1.21
TL14	0.6	0.6	17.9	53.2	27.6	4.06	0.73
TL15	0.0	0.0	17.3	53.8	28.8	4.12	0.67
TL16	0.6	0.6	22.4	46.2	30.1	4.04	0.78
TL17	0.0	4.5	19.2	46.2	30.1	4.02	0.82
TL18	1.3	3.8	25.6	42.3	26.9	3.90	0.89
TL19	0.6	3.8	20.5	47.4	27.6	3.97	0.83
TL20	0.6	4.5	20.5	50.0	24.4	3.93	0.83

Variable Mean						3.97	0.79

*Work Engagement*							

WE1	0.0	0.0	20.5	48.1	31.4	4.11	0.72
WE2	0.6	0.0	9.6	50.0	39.7	4.28	0.69
WE3	0.0	0.0	10.9	58.3	30.8	4.20	0.62
WE4	0.0	0.0	8.3	44.2	47.4	4.39	0.64
WE5	0.0	0.0	7.7	40.4	51.9	4.44	0.64
WE6	0.0	1.3	16.7	46.8	35.3	4.16	0.74
WE7	0.0	0.0	10.3	44.9	44.9	4.35	0.66
WE8	0.0	0.0	12.8	46.2	41.0	4.28	0.68
WE9	0.0	0.0	9.6	37.8	52.6	4.43	0.66
WE10	0.0	0.0	9.6	35.9	54.5	4.45	0.67
WE11	0.0	0.6	9.6	37.2	52.6	4.42	0.69
WE12	0.6	0.0	10.3	34.0	55.1	4.43	0.73
WE13	0.0	0.0	12.2	44.2	43.6	4.31	0.68
WE14	0.6	0.0	12.2	46.2	41.0	4.27	0.72
WE15	0.0	1.3	10.9	44.9	42.9	4.29	0.71
WE16	0.0	0.6	15.4	44.9	39.1	4.22	0.72
WE17	0.0	0.0	9.0	48.7	42.3	4.33	0.64

Variable Mean						4.32	0.68

*Organizational Citizenship behavior*							

OCBa1	0.0	0.0	6.4	46.2	47.4	4.41	0.61
OCBa2	0.0	0.0	9.6	50.6	39.7	4.30	0.64
OCBa3	0.0	0.0	5.8	48.7	45.5	4.40	0.60
OCBc1	0.0	0.0	7.7	49.4	42.9	4.35	0.62
OCBc2	1.3	0.6	19.2	49.4	29.5	4.05	0.79
OCBc3	1.9	3.8	32.7	41.0	20.5	3.74	0.89
OCBs1	0.0	3.2	21.2	48.7	26.9	3.99	0.78
OCBs2	0.6	6.4	34.0	39.1	19.9	3.71	0.88
OCBs3	3.2	10.9	42.3	25.6	17.9	3.44	1.01
OCBcs1	0.0	0.0	10.3	57.7	32.1	4.22	0.62
OCBcs2	0.0	0.0	12.8	55.1	32.1	4.19	0.64
OCBcs3	0.6	0.6	25.6	45.5	27.6	3.99	0.79
OCBtc1	0.0	4.5	19.2	44.2	32.1	4.04	0.83
OCBtc2	0.0	1.3	16.0	51.9	30.8	4.12	0.71
OCBtc3	0.0	0.0	9.6	44.2	46.2	4.37	0.65

Variable Mean						4.09	0.74

*Note:* PP (Proactive Personality), JS (Job Satisfaction), AC (Affective Commitment), OCBa (Organizational Citizenship behavior-altruism), OCBc (Organizational Citizenship behavior-Conscientiousness), OCBs (Organizational Citizenship behavior-Sportmanship), OCBcs (Organizational Citizenship behavior- Courtesy), OCBtc (Organizational Citizenship behavior- Civic Virtue).

**Table 3:** Multivariate normality.

Test	Kurtosis	c.r *multivariate*	Conclusion
*Multivariate normality*	837.41	56.90	c.r. is outside the range of ±1.96, thus the multivariate data are not normally distributed

## 2 Materials and Methods

Data were collected using purposive sampling technique, with the criteria for selecting respondents were prison officers whose job was to interact directly with the inmates, and work 24 h through work shifts. Data were entered and coded in SPSS v.24 software, while the preliminary test was carried out in SPSS v.24. For the main analysis, Amos v.24 was used as part of Structural Equation Modeling (SEM).

Multivariate normality refers to detecting the shape of the data distribution on a variable multivariately and its correspondence is a normal distribution. In SEM, the multivariate normality test is carried out with a critical ratio value (c.r.) in the multivariate kurtosis section, the value of c.r. This is also called the Z-value. If the Z-value is greater than the critical value, then the data distribution is not normal, on the other hand, if the Z-value is less than the critical value, the data distribution is normal. The critical value can be determined based on the significance level of 0.05 (5%) which is 1.96.

The results of the normality test showed a multivariate c.r of 56.90 which was outside the range of –1.96 to +1.96 at a significance level of 5%, so it can be concluded that the multivariate data were not normally distributed. This condition is actually not a problem because according to [5], Maximum Likelihood Estimation (MLE) in SEM is efficient and unbiased either the multivariate normality assumption is met or not, and has been proven to remain robust in the event of a violation of the normality assumption.

Next, Table 4 shows the results of the construct validity test conducted through convergent validity with the rule of thumb that a construct is said to meet convergent validity if the indicator on the construct has a standardized regression weight (lambda/factor loading) value above 0.50. Furthermore, Table 5 shows the construct reliability test that was checked using the construct reliability value. A construct is said to be reliable if the construct reliability value is greater than 0.70 or the Average Variance Extracted (AVE) value is above 0.50. Research [5] explains, the rule of thumb construct reliability value must be greater than 0.70, but the construct reliability value greater than 0.60 is still acceptable as long as each indicator has met convergent validity.

**Table 4:** Construct validity.

Variable	Dimension	Indicator	Factor Loading	Decision
Psychological Capital (X1)	–	PC1	0.781	Valid
		PC2	0.765	Valid
		PC3	0.813	Valid
		PC4	0.823	Valid
		PC5	0.858	Valid
		PC6	0.778	Valid
		PC7	0.783	Valid
		PC8	0.815	Valid
		PC9	0.713	Valid
		PC10	0.742	Valid
		PC11	0.777	Valid
		PC12	0.750	Valid

Transformational Leadership (X2)	–	TL1	0.714	Valid
		TL2	0.817	Valid
		TL3	0.784	Valid
		TL4	0.742	Valid
		TL5	0.842	Valid
		TL6	0.895	Valid
		TL7	0.875	Valid
		TL8	0.886	Valid
		TL9	0.874	Valid
		TL10	0.895	Valid
		TL11	0.876	Valid
		TL12	0.793	Valid
		TL13	0.652	Valid
		TL14	0.836	Valid
		TL15	0.836	Valid
		TL16	0.779	Valid
		TL17	0.790	Valid
		TL18	0.798	Valid
		TL19	0.745	Valid
		TL20	0.832	Valid

Work Engagement (Z)	–	WE1	0.727	Valid
		WE2	0.737	Valid
		WE3	0.773	Valid
		WE4	0.813	Valid
		WE5	0.785	Valid
		WE6	0.677	Valid
		WE7	0.887	Valid
		WE8	0.881	Valid
		WE9	0.870	Valid
		WE10	0.863	Valid
		WE11	0.850	Valid
		WE12	0.833	Valid
		WE13	0.862	Valid
		WE14	0.781	Valid
		WE15	0.799	Valid
		WE16	0.759	Valid
		WE17	0.846	Valid

Organizational Citizenship behavior (Y)	Altruism	OCBa1	0.884	Valid
		OCBa2	0.875	Valid
		OCBa3	0.906	Valid
	Conscientiousness	OCBc1	0.794	Valid
		OCBc2	0.674	Valid
		OCBc3	0.664	Valid
	Sportsmanship	OCBs1	0.699	Valid
		OCBs2	0.756	Valid
		OCBs3	0.730	Valid
	Courtesy	OCBcs1	0.830	Valid
		OCBcs2	0.889	Valid
		OCBcs3	0.722	Valid
	Civic Virtue	OCBtc1	0.775	Valid
		OCBtc2	0.884	Valid
		OCBtc3	0.843	Valid

*Note:* PP (Proactive Personality), JS (Job Satisfaction), AC (Affective Commitment), OCBa (Organizational Citizenship behavior-altruism), OCBc (Organizational Citizenship behavior-Conscientiousness), OCBs (Organizational Citizenship behavior-Sportsmanship), OCBcs (Organizational Citizenship behavior- Courtesy), OCBtc (Organizational Citizenship behavior- Civic Virtue).

**Table 5:** Construct reliability.

Construct	Construct Reliability	Average Variance Extracted	Decision
Psychological Capital (X1)	0.950	0.615	Reliable
Transformational Leadership (X2)	0.975	0.665	Reliable
Work Engagement (Z)	0.970	0.657	Reliable
Organizational Citizenship behavior (Y)			
Altruism	0.918	0.789	Reliable
Conscientiousness	0.755	0.509	Reliable
Sportsmanship	0.772	0.531	Reliable
Courtesy	0.856	0.667	Reliable
Civic Virtue	0.873	0.698	Reliable

**Table 6:** Summary of the direct effect hypotheses.

Structural relationship			Std. Estimate	P value(a)	Hypothesis
Psychological Capital	→	OCB	0.191	0.007⁎⁎	H_1_ accepted
Psychological Capital	→	Work Engagement	0.351	0.006⁎⁎	H_2_ accepted
Transformational Leadership	→	OCB	0.369	0.025*	H_3_ accepted
Transformational Leadership	→	Work Engagement	0.463	0.019*	H_4_ accepted
Work Engagement	→	OCB	0.432	0.002⁎⁎	H_5_ accepted

⁎ . Significant at the 0.05 level.

⁎⁎ . Significant at the 0.01 level n.s. Not significant.

(a) p-value has been calculated based on MLE bootstrapping.

**Fig. 1:**
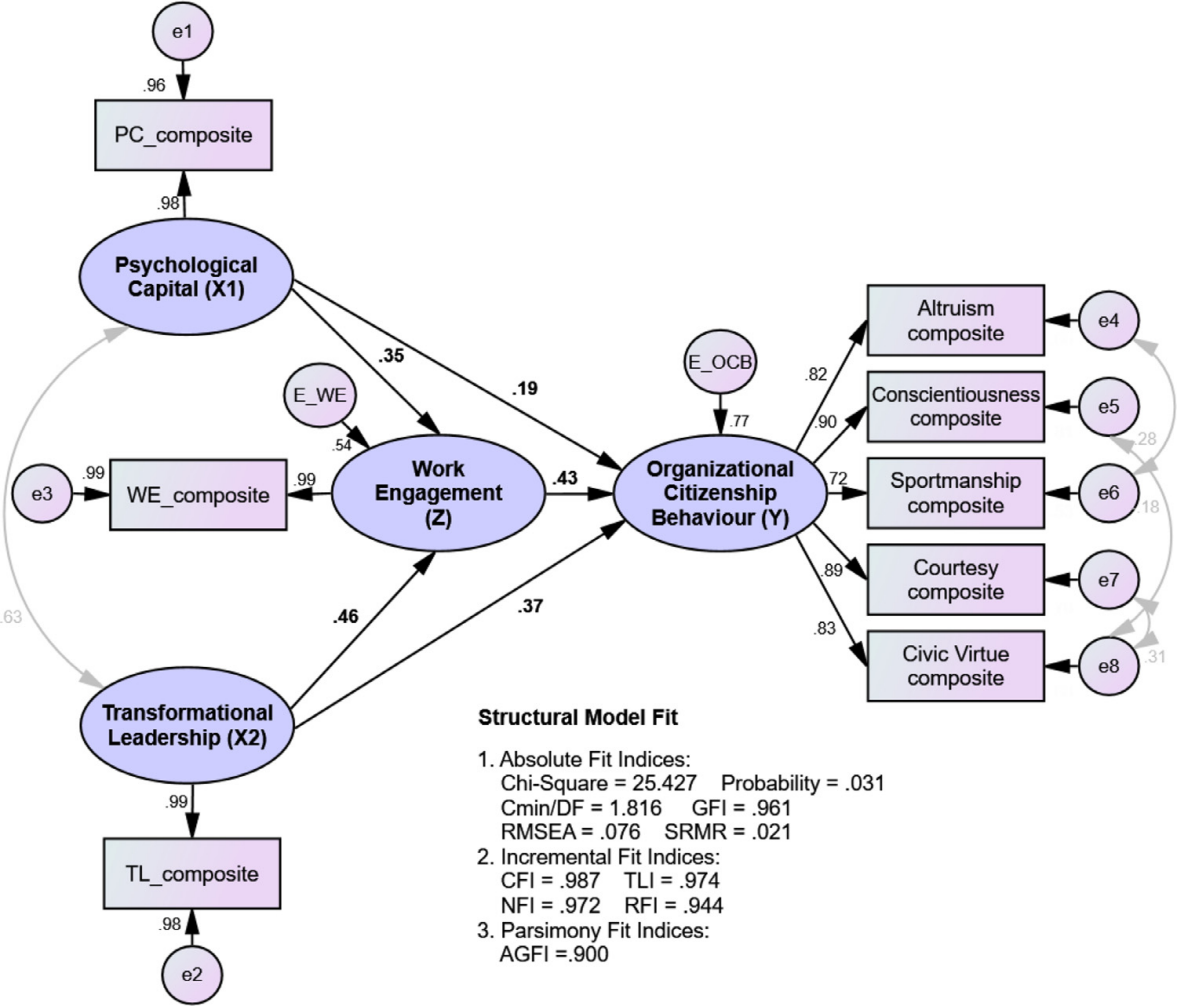
Assesing the structural model.

The structural model stage begins with an evaluation of the structural model fit (goodness of fit) whose role is to ensure that the developed model is consistent with the data. The estimation results are presented in Fig. 1 below:

After getting 156 responses from respondents according to the research criteria, then all samples were processed with v.24 software to analyze validity, reliability, structural models, direct effect hypotheses, and indirect effect hypotheses.

Structural model analysis is done by examining the estimated parametric relationship between variables that represent each theoretical hypothesis on the direct effect path. This assumption can be accepted if the path parameter is significant in the predicted effect, meaning that the path parameter must be greater than zero for the positive direction and less than zero for the negative direction [5]. Furthermore, in the structural relationships test, hypothesis testing is carried out for the influence between variables, using the critical ratio (CR) value and the probability value (p-value). Whether or not there is a significant effect between variables using the provisions if the CR value 1.96 or the p-value 5% significance level, then it is decided that there is a significant effect between these variables. Likewise, the indirect effect path has the same provisions.

After testing the significance of the mediation effect, the final step is to know the nature of the mediation (described on Table 7). Detecting the nature of mediation can be seen from the effect of mediation, if the direct effect of the exogenous variable on the endogenous variable is significant, and the indirect effect of the mediating variable also passes through the significant path, it is said to be partially mediation [6]. On the other hand, if the direct effect of the exogenous variable on the endogenous variable is not significant, while the indirect effect is through the mediating variable through a significant path, then it is said to be fully mediation or perfect mediation. Table 7 shows the role of employee involvement at work in order to support employee psychology and leadership style in influencing organizational citizenship behavior.

**Table 7:** Summary of the indirect effect hypotheses.

Structural relationship	Std. Estimate	P-value(a)	Hypothesis	Type of mediator
Psychological Capital →Work Engagement →Organizational Citizenship behavior	0.152	0.001⁎⁎	H_6_ accepted	Partially mediation

Transformational Leadership→ Work Engagement →Organizational Citizenship behavior	0.200	0.003⁎⁎	H_7_ accepted	Partially mediation

*Significant at the 0.05 level.

⁎⁎ Significant at the 0.01 level n.s. Not significant.

(a) calculated based on MLE bootstrapping.

## Ethics Statement and Informed Consent

This is a non-interventional research project. No ethical permission is necessary, according to the Research Ethics Committee of Universitas Airlangga. Informed consent has been acquired from all participants of the study.

## CRediT authorship contribution statement

**Ahmad Rizki Sridadi:** Conceptualization, Project administration. **Anis Eliyana:** Conceptualization, Data curation, Resources. **Desynta Rahmawati Gunawan:** Conceptualization, Investigation. **Muhammad Danang Kurniawan:** Data curation, Visualization. **Alvin Permana Emur:** Investigation, Validation. **Zaleha Yazid:** Supervision.

## Declaration of Competing Interest

The authors state that no financial or personal conflict of interest is deemed to affect the work reported in this article.

## Data Availability

The Mediating Role of Work Engagement: A Survey Data on Organizational Citizenship Behavior (Original data) (Mendeley Data).
